# Antimicrobial resistance in *Neisseria gonorrhoeae* in nine sentinel countries within the World Health Organization Enhanced Gonococcal Antimicrobial Surveillance Programme (EGASP), 2023: a retrospective observational study

**DOI:** 10.1016/j.lanwpc.2025.101663

**Published:** 2025-08-21

**Authors:** Ismael Maatouk, Phiona Vumbugwa, Thitima Cherdtrakulkiat, Lon Say Heng, Irving Hoffman, Noel Palaypayon, Francis Kakooza, Rossaphorn Kittiyaowamarn, Peter Kyambadde, Monica M. Lahra, Pham Thi Lan, Venessa Maseko, Mitch Matoga, Anna Machiha, Etienne Müller, Thuy Thi Phan Nguyen, Le Huu Doanh, Vichea Ouk, Vivi Setiawaty, Sonia B. Sia, Teguh S. Hartono, Mot Virak, Nguyen Thi Thuy Van, Magnus Unemo, Teodora Wi, WHO EGASP Study Group, Lon Say Heng, Vichea Ouk, Mot Virak, Phal Kun Mom, Serongkea Deng, Vivi Setiawaty, Endang Lukitosari, Nurhalina Afriana, Verawati Sulaiman, Teguh S. Hartono, Maria Laurensia, Ni Luh Putu Pitawati, Mitch Matoga, Irving Hoffman, Robert Krysiak, Jane Chen, Naomi Bonongwe, Claightone Chirombo, Edward Jere, James Kapha, Sonia B. Sia, Manuel C. Jamoralin, Marietta Lagrada, June Gayeta, Jaywardeen Abad, Noel Palaypayon, Diana Lim, Iftizar N. Haron, Joseph Carlo Sangco, Felyrose Fuertes, August Cesar Abrajano, Ruby Rusia-Uy, Christine Ivy Paula S. Agtuca, Ma. Theresa A. Fedoc-Minguito, Louwela A. Jerusalem, Venessa Maseko, Etienne Müller, Lindy Gumede, Portia Baloyi, Rossaphorn Kittiyaowamarn, Natnaree Girdthep, Porntip Paopang, Pongsathorn Sangprasert, Thitima Cherdtrakulkiat, Jaray Tongtoyai, Francis Kakooza, Peter Kyambadde, Emmanuel Mande, Martha Nakasi, Le Huu Doanh, Pham Thi Lan, Pham Quynh Hoa, Pham Dieu Hoa, Thuy Thi Phan Nguyen, Hao Trong Nguyen, Nhi Thi Uyen Pham, Phuong Thi Thanh Nguyen, Nguyen Thi Thuy Van, Francis Slaughter, Anna Machiha, Owen Mugurungi, Agnes Juru, Tatenda Ngorima, Lucia Sisya, Kudzai Takarinda, Andrew Tarupiwa, Muchaneta Mugabe, Mkhokheli Ngwenya, Precious Paidamoyo Andifasi, Monica Lahra, Sebastian van Hal, Magnus Unemo, Daniel Golparian, Susanne Jacobsson, Daniel Schröder, Teodora Wi, Ismael Maatouk, Phiona Vumbugwa

**Affiliations:** a Global HIV, Hepatitis and STI Programmes, World Health Organization (WHO), Geneva, Switzerland; b Bangrak STIs Center, Division of AIDS and STIs, Department of Disease Control, Ministry of Public Health, Bangkok, Thailand; c National Center for HIV/AIDS, Dermatology and Sexually Transmitted Diseases, Phnom Penh, Cambodia; d University of North Carolina (UNC) Project Malawi, Lilongwe, Malawi; e Department of Health, Research Institute for Tropical Medicine, Manila, the Philippines; f Infectious Diseases Institute, Makerere University College of Health Sciences, Kampala, Uganda; g Sexually Transmitted Infections Program, Ministry of Health, Kampala, Uganda; h WHO Collaborating Centre for Sexually Transmitted Infections and Antimicrobial Resistance, New South Wales Health Pathology, Microbiology, Randwick, NSW, Australia; i National Hospital of Dermatology and Venereology, and Hanoi Medical University, Viet Nam; j Centre for HIV and STIs, National Institute for Communicable Diseases, National Health Laboratory Service, Johannesburg, South Africa; k Sexually Transmitted Infections, Ministry of Health, Harare, Zimbabwe; l Ho Chi Minh City Hospital of Dermatology and Venereology, Ho Chi Minh City, Viet Nam; m Sulianti Saroso Infectious Disease Hospital, Jakarta, Indonesia; n Laboratory of the National Institute of Public Health, Phnom Penh, Cambodia; o WHO Country Office, Hanoi, Viet Nam; p WHO Collaborating Centre for Gonorrhoea and Other Sexually Transmitted Infections, Örebro University, Örebro, Sweden; q University College London, London, United Kingdom

**Keywords:** Antimicrobial resistance, *Neisseria gonorrhoeae*, Gonorrhoea, Surveillance, Enhanced Gonococcal Antimicrobial Surveillance Programme (EGASP)

## Abstract

**Background:**

The global spread of antimicrobial resistance (AMR) in *Neisseria gonorrhoeae* threatens empiric single-dose gonorrhoea treatment. Enhanced global AMR surveillance is imperative. We report i) gonococcal antimicrobial susceptibility and resistance data from 2023 in the World Health Organization Enhanced Gonococcal Antimicrobial Surveillance Programme (WHO EGASP) in the WHO Western Pacific Region (Cambodia, the Philippines, Viet Nam), Southeast Asian Region (Indonesia, Thailand), and African Region (Malawi, South Africa, Uganda, Zimbabwe), and ii) metadata of the gonorrhoea patients.

**Methods:**

In 2023, WHO EGASP included men with urethral discharge (n = 3498) and gonococcal isolates (n = 2491). Minimum inhibitory concentrations (MICs, mg/L) values were determined for ceftriaxone, cefixime, azithromycin, gentamicin, and ciprofloxacin using Etest (bioMérieux). Breakpoints from the European Committee on Antimicrobial Susceptibility Testing (EUCAST) were applied. Clinical and epidemiological variables associated with AMR isolates were assessed using univariable and multivariable logistic regression analyses of odds ratios.

**Findings:**

Overall, 3.8% (95% confidence interval (95% CI) 3.1–4.6%; 95/2487), 8.9% (95% CI 7.9–10.1%; 222/2484), 3.6% (95% CI 2.9–4.4%; 89/2487), and 95.3% (95% CI 93.2–97.5%; 1801/1890) of isolates were resistant to ceftriaxone, cefixime, azithromycin, and ciprofloxacin, respectively. All the ceftriaxone-resistant isolates were from Cambodia (15.3% (95% CI 11.5–20.1%), 42/274) and Viet Nam (20.4% (95% CI 15.9–25.7%), 53/260). In univariable analysis, ceftriaxone resistance was associated with travelling within the country during previous 30 days (OR 4.66, 95% CI 3.06–7.16; *p* < 0.001), and this association remained in multivariable analysis (aOR 4.12, 95% CI 2.65–6.65; *p* < 0.001).

**Interpretation:**

Resistance to ceftriaxone, cefixime, and azithromycin is a major global concern, and expanded and improved resistance surveillance is essential. The WHO EGASP has been substantially expanded in the recent years. Additionally, resistance breakpoints have been harmonised and test-of-cure, whole-genome sequencing, and extragenital sampling implemented, where feasible. Novel antimicrobials for gonorrhoea treatment are critical; zoliflodacin and gepotidacin are promising.

**Funding:**

10.13039/100004423WHO, 10.13039/100004417Global Fund.

**Research in context**

**Evidence before this study**

The global prevalence of gonorrhoea is high and antimicrobial resistance (AMR) in *Neisseria gonorrhoeae* causes serious difficulties in the treatment of gonorrhoea. Recent publications from several countries, especially in Asia, have described higher levels of resistance to the last option for empiric first-line gonorrhoea treatment, ceftriaxone. Appropriate global gonococcal AMR surveillance is essential. We searched PubMed for original articles using the terms “*N*eisseria *gonorrhoeae*” AND “surveillance” AND “global” AND “antimicrobial susceptibility” OR “antimicrobial resistance” up to March 1, 2025. Three World Health Organization (WHO) Gonococcal Antimicrobial Surveillance Programme (GASP) original articles surveying global gonococcal AMR were found. However, the most recent global AMR data were from 2018, and the main conclusions of these papers included that the global gonococcal AMR surveillance needs to be substantially enhanced, standardised and quality-assured, and combined with epidemiological data of the gonorrhoea patients.

**Added value of this study**

International programmes surveying gonococcal AMR are limited. We report international, standardised and quality-assured gonococcal antimicrobial susceptibility/resistance data from 2023 in the WHO Enhanced GASP (WHO EGASP) including nine sentinel countries in the WHO Western Pacific Region, Southeast Asian Region, and African Region. Several of these countries lacked or had limited gonococcal AMR surveillance prior to inclusion in the WHO EGASP. We additionally report demographical, behavioural, and clinical data of the corresponding gonorrhoea patients, and examine associations between gonococcal AMR and these metadata. The levels of resistance to ceftriaxone, cefixime, azithromycin, and ciprofloxacin was substantial, i.e., 4% (95/2487), 9% (222/2484), 4% (89/2487), and 95% (1801/1890), respectively. The ceftriaxone-resistant isolates (n = 95) were all from Cambodia (15%, 42/274) or Viet Nam (20%, 53/260). In total, 75% (71/95) of the ceftriaxone-resistant isolates had a ceftriaxone MIC of 0.25 mg/L, i.e., just above the ceftriaxone resistance breakpoint. The vast majority of the resistance to also cefixime and azithromycin was detected in Cambodia and Viet Nam, respectively. The levels of resistance to ceftriaxone and cefixime in Cambodia and Viet Nam in the present study are among the highest resistance levels described globally. In univariable analysis, ceftriaxone resistance was associated with travelling within the country; and cefixime and azithromycin were associated with travelling within the country and men who had sex with women only. All these associations remained in the multivariable analysis.

**Implications of all the available evidence**

WHO EGASP provides standardised and quality-assured gonococcal AMR monitoring, combined with demographical, behavioural, and clinical data of the gonorrhoea patients, in sentinel countries in three WHO regions. The WHO EGASP describes high resistance to ceftriaxone in Cambodia and Viet Nam, associated with intra-country travelling, and ceftriaxone-resistant strains from these countries have been spreading to, for example, Europe, which emphasises the importance of increased gonococcal AMR surveillance in these Asian countries. The WHO EGASP data have additionally informed revisions of the national treatment guidelines in Cambodia and Viet Nam. Notably, the WHO EGASP is continuously improving and expanding. Ultimately, novel antimicrobials for the treatment of gonorrhoea are essential; zoliflodacin and gepotidacin have shown non-inferiority compared to a ceftriaxone plus azithromycin regimen for the treatment of uncomplicated gonorrhoea in phase 3 randomised controlled clinical trials.

## Introduction

In 2020, the World Health Organization (WHO) estimated 82.4 million new global gonorrhoea cases each year among adults (aged 15–49 years), which cause an unknown number of serious complications such as pelvic inflammatory disease, ectopic pregnancy, infertility, and increased transmission of HIV.^1^^,^^2^ The highest gonorrhoea incidences have for many decades been estimated in the WHO Western Pacific Region, African Region, and Southeast Asian Region.^3^

*Neisseria gonorrhoeae* has developed or acquired resistance to all antimicrobials introduced for treatment of gonorrhoea and, in recent decade, resistance to the last options for empirical first-line treatment, ceftriaxone monotherapy or ceftriaxone-azithromycin dual therapy, has emerged internationally.^2^^,^^4^, ^5^, ^6^, ^7^, ^8^, ^9^, ^10^, ^11^, ^12^, ^13^, ^14^, ^15^, ^16^, ^17^, ^18^, ^19^ Gonococcal strains with resistance to both ceftriaxone and azithromycin have been detected in many countries and untreatable gonorrhoea cases with standard treatment may soon become a reality.^10^^,^^13^, ^14^, ^15^, ^16^, ^17^ Most ceftriaxone-resistant gonococcal strains have been detected in the WHO Western Pacific Region or in other regions after patients being infected in Asia.^2^^,^^5^^,^^6^^,^^9^, ^10^, ^11^, ^12^, ^13^, ^14^, ^15^, ^16^, ^17^, ^18^, ^19^

International surveillance of gonorrhoea cases and antimicrobial resistance (AMR) in *N. gonorrhoeae* are critical to improve our understanding of global gonorrhoea epidemiology, clinical management and emergence and spread of antimicrobial-resistant *N. gonorrhoeae* strains. The WHO global Gonococcal Antimicrobial Surveillance Programme (GASP) was revitalised in 2009 and it has subsequently been further strengthened.^5^^,^^18^^,^^19^ The WHO GASP has provided important AMR data used to refine national, international and global treatment guidelines. However, WHO GASP has limitations such as suboptimal standardisation and quality assurance, and lack of metadata of the gonorrhoea patients.^5^ The WHO Enhanced GASP (EGASP),^10^^,^^11^^,^^13^^,^^20^ focussing on representative sentinel countries globally, was developed to address many of the limitations in WHO GASP. In accordance with the WHO EGASP General Protocol and the WHO EGASP Supplementary Protocol,^21^ countries use standardised and quality assured protocols for recruitment of participants, gonococcal culture and AMR testing; collect demographic, behavioural and, clinical data of the gonorrhoea patients; and aims to early detect and timely report *N. gonorrhoeae* strains with elevated minimum inhibitory concentrations (MICs) of the internationally recommended treatments for gonorrhoea. WHO EGASP implementation aligns with both the WHO global action plan to control the spread and impact of AMR in *N. gonorrhoeae* and the WHO global action plan on AMR.^22^^,^^23^ By December 2023, WHO EGASP had been expanded to include nine representative sentinel countries in the WHO Western Pacific Region (Cambodia,^10^^,^^13^ the Philippines, Viet Nam^11^), Southeast Asian Region (Indonesia, Thailand^20^), and African Region (Malawi, South Africa, Uganda, Zimbabwe). Furthermore, Argentina Brazil, Côte d'Ivoire, India, and Qatar were in different phases of planning their WHO EGASP implementation. The WHO EGASP effectively supplements the WHO GASP and it is imperative to elucidate and monitor global gonorrhoea epidemiology and gonococcal AMR, including emerging AMR.

We report i) *N. gonorrhoeae* antimicrobial susceptibility/resistance data from nine representative WHO EGASP sentinel countries within the WHO Western Pacific Region (Cambodia, the Philippines, Viet Nam), Southeast Asian Region (Indonesia, Thailand), and African Region (Malawi, South Africa, Uganda, Zimbabwe) in 2023, and ii) demographic, behavioural, and clinical data of the gonorrhoea patients.

## Methods

### Surveillance settings

Thirty-eight clinical sentinel sites and 17 laboratories in the nine countries participated and 37 clinical sites (16 laboratories) collected urethral discharge episodes and gonococcal isolates in WHO EGASP in 2023 (Appendix p1–2: Table S1). Notably, some data in the present study have been previously published. Accordingly, the genomic sequences and summarised levels of resistance to ceftriaxone, cefixime and azithromycin for 249 of the 260 isolates from Viet Nam have been published in a brief Correspondence.^11^ The resistance to ceftriaxone, cefixime, azithromycin, ciprofloxacin, and gentamicin in 108 of the 274 isolates, including the genomic sequences of 72 isolates, from Cambodia have also been previously published.^10^^,^^13^ Due to the exceedingly high levels of resistance to ceftriaxone and cefixime identified in these first results, it was considered urgent and most ethical to rapidly publish these, i.e., to inform the scientific community and other public health organisation, and to initiate a revision of the national gonorrhoea treatment guidelines in Cambodia and Viet Nam. No WHO EGASP 2023 data have been published from the other seven countries (2858 urethral discharge episodes, 1957 gonococcal isolates).

### Participant enrolment and sampling

WHO EGASP systematically includes men attending participating sentinel clinics with a suspected urogenital gonorrhoea episode (presence of urethral discharge). The inclusion criteria are as follows: men with urethral discharge, attending a participating clinic for the first time for the current suspected urogenital gonorrhoea episode, older than the legal age of consent in the country, and able and willing to provide consent and a urethral specimen for testing. The exclusion criteria are: attending the clinic to follow up of a previously treated urogenital gonorrhoea episode and do not give informed consent to participate. A urethral swab is sampled from each consenting man included in WHO EGASP. In some sentinel clinics, all urethral swab samples are cultured, while in other clinics only urethral swab samples that show intracellular Gram-negative diplococci in microscopy are cultured (Table 1). The aim is to recruit a minimum of 150–200 men, i.e., to obtain a minimum sample size of 100 gonococcal isolates per sentinel country or site.^21^

**Table 1:** WHO EGASP general data, 2023.

	Cambodia	Indonesia	Malawi	Philippines	South Africa	Thailand	Uganda	Viet Nam	Zimbabwe	Overall
Year of EGASP initiation	2022	2023	2023	2018	2023	2015	2022	2023	2023	
No. of clinical sentinel sites	10	4	1	7	3	4	4	2	3	**38**
No. of reference laboratories	1	1	1	4	1	4	2	2	1	**17**
Included EGASP patients with										
1 UD episode	351	126	126	746	522	623	417	258	107	**3276**
2 UD episodes	12	0	0	20	3	55	3	2	0	**95**
≥3 UD episodes	1	0	0	0	0	9	0	0	0	**10**
Included EGASP patients	364	126	126	766	525	687	420	260	107	**3381**
Included UD episodes	380	126	126	786	528	762	423	260	107	**3498**
NG culture positive (% of included UD episodes)	274 (72)	105 (83)^a^	122 (97)^a^	700 (89)^a^	344 (65)	375 (49)	298 (70)	260 (100)^a^	24 (22)	**2502 (72)**
NG AMR tested (% of NG isolates)	274 (100)	105 (100)	122 (100)	700 (100)	341 (99.1)	373 (99.5)	298 (100)	260 (100)	18 (75)	**2491 (99.6)**

WHO = World Health Organization; EGASP = Enhanced Gonococcal Antimicrobial Surveillance Programme; No. = number; UD = Urethral discharge; NG = *Neisseria gonorrhoeae*; AMR = antimicrobial resistance.

a Some countries cultured samples from males with urethral discharge when intracellular Gram-negative diplococci were found in microscopy.

### Laboratory procedures

Gonococcal culture, including *N. gonorrhoeae* species identification, was performed in accordance with routine microbiological diagnostic procedures.^24^ MICs (mg/L) were determined for the mandatory antimicrobials; ceftriaxone, cefixime, azithromycin and for the optional antimicrobials; ciprofloxacin (in seven countries) and gentamicin (in seven countries) using Etest (bioMérieux, Marcy l'*Etoile*, France), in accordance with the manufacturer's instructions. Clinical breakpoints from the European Committee on Antimicrobial Susceptibility Testing (EUCAST)^25^ were used for ceftriaxone (susceptible MIC ≤ 0.125 mg/L, resistant MIC > 0.125 mg/L), cefixime (susceptible MIC ≤ 0.125 mg/L, resistant MIC > 0.125 mg/L), and ciprofloxacin (susceptible MIC ≤ 0.032 mg/L, resistant MIC > 0.064 mg/L). For azithromycin, due to the lack of clinical breakpoints, the EUCAST epidemiological cut-off (ECOFF) value (MIC > 1 mg/L) was used to indicate resistance.^25^ Because no clinical breakpoints or ECOFF value exist for gentamicin, only the MICs are described. Isolates resistant to ceftriaxone, cefixime and azithromycin were retested for confirmation and reported timely to WHO. Notably, one gonococcal isolate per gonorrhoea episode was reported. For quality control of culture diagnostics and AMR testing, the *N. gonorrhoeae* reference strain ATCC 49226 and the 2024 WHO gonococcal reference strains (WHO L and U are mandatory, but also additional ones are used in some countries) were used.^26^

### Standardised data collection, management, and validation

Sentinel clinics assigned unique participant identification and EGASP identification numbers and collected demographic, behavioural, and clinical data of the gonorrhoea participants using a standardised paper-based or electronic data collection form. The data were then transmitted to the reference laboratory with the EGASP specimen(s). An EGASP coordinator used the standardised EGASP data entry form to deduplicate, check for completeness and accuracy of country surveillance data before submitting to the EGASP module in the IT platform for the WHO Global Antimicrobial Resistance and Use Surveillance System (GLASS) within two months after data validation. All clinical and laboratory data were entered directly into password-protected and encrypted tablets/computers using either Excel, ODK, Laboratory Information System (LIS), Microsoft Access and converted using the RStudio program, or WHONET Platform. All paper-based and electronic data are stored securely, and only authorised personnel have access.

### Data analysis

Statistical analysis was performed using R v4.5.0. Categorical patient variables used were as follows: age-group (<25 and ≥25 years); antibiotic use in the past 2 weeks; history of travel, sexual history, and number of sexual partners during past 30 days; HIV status; and concurrent STIs. Associations between these patient variables and AMR isolates were assessed using univariable and multivariable logistic regression analyses, crude and adjusted odds ratios (ORs), respectively, 95% confidence intervals (CI) and *p*-values. For sufficient cell counts (n ≥ 10), ORs and 95% CIs were calculated and Pearson's χ^2^ test was used to measure if these ORs differed significantly from 1. Observations missing data for the variables in the univariable or multivariable models were excluded from the analyses. For each antimicrobial, variables significantly associated with resistance in univariable models were further analysed in multivariable models, with adjustment for a priori defined potential confounders. Specifically, models included history of internal travel and sexual history as exposures of interest and adjusted for antibiotic use in the past two weeks, concurrent STI diagnosis, and the alternate exposure (i.e., sexual history or travel) as appropriate. Adjusted ORs (aORs) with 95% confidence intervals (CIs) were estimated. Statistical significance for all tests was assumed as a two-sided *p*-value of <0.05 for tests.

### Ethics approval

The WHO EGASP protocols were approved (Protocol ID: EGASPprotocolGen, Date: 20 May, 2020) by the Research Ethics Review Committee of the WHO, Geneva, Switzerland; ethics boards in the EGASP countries, where required; and confirmed as standard clinical practice and public health surveillance. Nevertheless, informed oral or written consent was obtained from each participant and the participants were assigned unique patient and EGASP identification numbers, to maintain privacy and confidentiality. Data was also stored in a password protected server, accessible only to authorised staff with unique credentials.

### Role of the funding source

The funders had no role in the study design, data collection, analysis, interpretation, or writing of the report.

## Results

### WHO EGASP participants

In 2023, 3498 urethral discharge episodes in men (n = 3381) were included. Of these, 22% (786/3498) were from the Philippines, 22% (762/3498) from Thailand, 15% (528/3498) from South Africa, 12% (423/3498) from Uganda, 11% (380/3498) from Cambodia, 7% (260/3498) from Viet Nam, 4% (126/3498) from Indonesia, 4% (126/3498) from Malawi, and 3% (107/3498) from Zimbabwe (Table 1).

### Demographic, behavioural, and clinical data of the gonorrhoea patients

Most males were from 25 to 34 years (38%, 1327/3498), followed by 18–24 years (35%, 1225/3498), and the median age was 27 years (interquartile range (IQR): 22–34 years; range: 12–93 years) (Appendix p3: Table S2). Antibiotic use within the past two weeks was reported in 11% (385/3498) of included urethral discharge episodes, with higher proportions in Thailand (22%, 168/762), Indonesia (19%, 24/126), and Uganda (18%, 77/423). Travel history was reported in all countries except South Africa and Thailand. Briefly, 24% (533/2208) of all participants in reporting countries reported travelling during the recent 30 days (22% (490/2208) within country only, 1.6% (35/2208) internationally, and 0.4% (8/2208) both within country and internationally). Viet Nam reported the highest frequency on travel (71%, 184/260), however, 68% (178/260) of participants were travelling within the country only. Number of sexual partners in the previous 30 days was reported in all countries except Thailand. Overall, 52% (1420/2736) of participants reported having one sexual partner, and 45% (1225/2736) had two or more sexual partners (Appendix p3: Table S2).

Data on sexual history and practices was not reported in Malawi and Uganda (Appendix p4: Table S3). In the seven reporting countries, 71% (2085/2949) of participants reported having sex with women only, 23% (675/2949) reported having sex with men only, and 6% (176/2949) reported having sex with both men and women during previous 30 days. In countries where sexual practices were reported (Cambodia, Indonesia, Philippines, Viet Nam, and Zimbabwe), vaginal intercourse was most common (64%, 1064/1659); while oral and anal sex was reported in 35% (577/1659) and 39% (601/1557) of cases, respectively. Among the 6% (139/2208) of participants who self-reported living with HIV, Zimbabwe, Indonesia, and Cambodia had the highest percentages. However, this variable was unknown for 79% (1741/2208) of cases in reporting countries and Thailand and South Africa did not report on this variable. In general, most EGASP participants were not tested for many other STIs (Appendix p4: Table S3).

### Culture and antimicrobial susceptibility testing of *N. gonorrhoeae*

Of the 3498 included urethral discharge episodes, 72% (2502/3498) were *N. gonorrhoeae* culture positive. Of these 2502 gonococcal isolates, 2491 were available for antimicrobial susceptibility testing by Etest (Table 1). The gonococcal culture positivity rate ranged from 22% (24/107, Zimbabwe) to 100% (260/260, Viet Nam).

### Antimicrobial susceptibility/resistance

Overall, 3.8% (95% CI 3.1–4.6%; 95/2487), 8.9% (95% CI 7.9–10.1%; 222/2484), 3.6% (95% CI 2.9–4.4%; 89/2487), and 95.3% (95% CI 93.2–97.5%; 1801/1890) of isolates were resistant to ceftriaxone, cefixime, azithromycin, and ciprofloxacin, respectively (Table 2). All the ceftriaxone-resistant isolates were cultured in the WHO Western Pacific Region countries Cambodia (15%, 42/274) and Viet Nam (20%, 53/260), and these countries had substantially higher resistance to also cefixime (53% (144/274) and 30% (78/260), respectively) and azithromycin (21% (58/274) and 7% (18/260), respectively). In Cambodia, the proportion of isolates with resistance to ceftriaxone, cefixime plus azithromycin was 9% (26/274). In Viet Nam, the proportion of isolates with resistance to all these three antimicrobials was 2% (4/260). South Africa and Thailand showed 3% (9/341) and 1% (4/373) resistance to azithromycin, respectively. In Indonesia, Malawi, the Philippines, Uganda, and Zimbabwe, all isolates were susceptible to ceftriaxone, cefixime, and azithromycin. Ciprofloxacin resistance was high in all seven countries where it was surveyed (95% (1801/1890), range: 76–100%). Of 2172 isolates tested in seven countries, all isolates except two Vietnamese isolates (0.1%, 2/2172) had gentamicin MICs of ≤16 mg/L (Table 2).

**Table 2:** Antimicrobial resistance in *Neisseria gonorrhoeae* isolates in nine WHO EGASP countries, 2023.

	Ceftriaxone No. (%, 95% CI)	Cefixime No. (%, 95% CI)	Azithromycin No. (%, 95% CI)	Ciprofloxacin No. (%, 95% CI)	**MIC gentamicin >16 mg/L (%, 95% CI)**
Cambodia (n = 274)	42 (15.3, 11.5–20.1)	144 (52.6, 46.6–58.4)	58 (21.2, 16.7–26.4)	273 (99.6, 98.0–99.9)	0 (0, 0–1.4)
Indonesia (n = 105)	0 (0, 0–3.5)	0 (0, 0–3.5)	0 (0, 0–3.5)	102 (97.1, 91.9–99.0)	0 (0, 0–3.5)
Malawi (n = 122)	0 (0, 0–3.1)	0 (0, 0–3.1)	0 (0, 0–3.1)	94 (77.0, 68.8–83.6)	0 (0, 0–3.1)
Philippines (n = 700)	0 (0, 0–0.5)	0 (0, 0–0.5)	0 (0, 0–0.5)	656 (93.7, 91.7–95.3)	0 (0, 0–0.5)
South Africa (n = 341)	0 (0, 0–1.1)	0 (0, 0–1.1)	9 (2.6, 1.4–4.9)	ND	0 (0, 0–1.1)
Thailand (n = 373)	0 (0, 0–1.0)	0 (0, 0–1.0)	4 (1.1, 0.4–2.7)	365 (97.6, 95.4–99.8)	0 (0, 0–1.0)
Uganda (n = 298)	0 (0, 0–1.3)	0 (0, 0–1.3)	0 (0, 0–1.3)	298 (100, 98.7–100)	ND
Viet Nam (n = 260)	53 (20.4, 15.9–25.7)	78 (30.0, 24.8–35.8)	18 (6.9, 4.4–10.7)	ND	2 (0.8, 0.2–2.8)
Zimbabwe (n = 18)	0 (0, 0–17.6)	0 (0, 0–17.6)	0 (0, 0–17.6)	13 (72.2, 49.1–87.5)	ND
Overall (n = 2491)^a^	95 (3.8, 3.1–4.6)	222 (8.9, 7.9–10.1)	89 (3.6, 2.9–4.4)	1801 (95.3, 93.2–97.5)	2 (0.1, 0–0.3)

WHO = World Health Organization; EGASP = Enhanced Gonococcal Antimicrobial Surveillance Programme; n = number of isolates (one isolate per gonorrhoea episode); No. = number; ND = not determined.

a Susceptibility to ceftriaxone, cefixime, azithromycin, ciprofloxacin, and gentamicin was tested for 2487, 2484, 2487, 1890, and 2172 isolates, respectively, using Etest (bioMérieux, Marcy l'*Etoile*, France).

**Fig. 1:**
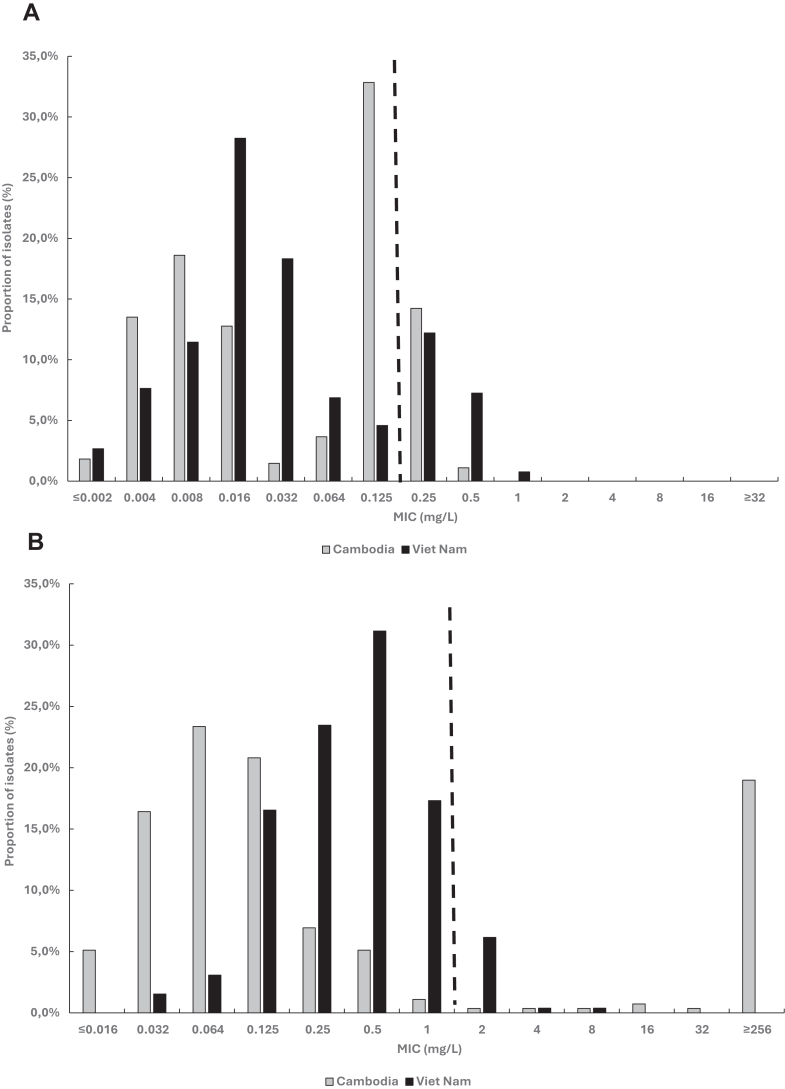
Ceftriaxone (A) and azithromycin (B) MIC distributions for *N. gonorrhoeae* isolates from Cambodia and Viet Nam, WHO EGASP, 2023. Black dashed lines depict the EUCAST clinical resistance breakpoints.^25^

Overall, the ceftriaxone MICs ranged from ≤0.002 mg/L to 1 mg/L, and the ceftriaxone MIC distributions for gonococcal isolates from Cambodia and Viet Nam are illustrated in Fig. 1A. Notably, the ceftriaxone-resistant isolates (Viet Nam: 53 and Cambodia: 42) had ceftriaxone MICs of 0.25 mg/L (n = 71), 0.5 mg/L (n = 22), and 1 mg/L (n = 2). Furthermore, the MICs of azithromycin ranged from ≤0.016 mg/L to ≥256 mg/L, and the azithromycin MIC distributions for gonococcal isolates from Cambodia and Viet Nam are shown in Fig. 1B.

In univariable analysis, ceftriaxone resistance was associated with travelling within the country during previous 30 days (OR 4.66, 95% CI 3.06–7.16; *p* < 0.001) (Appendix p5: Table S4); cefixime was associated with travelling within the country (OR 4.11, 95% CI 3.07–5.50; *p* < 0.001) and men who had sex with women only (OR 2.92, 95% CI 2.05–4.29; *p* < 0.001) (Appendix p6: Table S5); and azithromycin resistance was associated with travelling within the country (OR 1.99, 95% CI 1.22–3.18; *p* < 0.001) and men who had sex with women only (OR 2.57, 95% CI 1.39–5.20; *p* < 0.001) (Appendix p7: Table S6). In multivariable analysis (adjusted models), history of travelling within the country in the previous 30 days remained associated with increased odds of resistance to all three antimicrobials. The strongest association was observed for ceftriaxone resistance (aOR 4.12, 95% CI 2.65–6.65; *p* < 0.001), followed by cefixime resistance (aOR 3.75, 95% CI 2.62–5.37; *p* < 0.001), and azithromycin resistance (aOR 1.73, 95% CI 1.03–2.91; *p* = 0.043). Furthermore, sexual history remained associated with resistance to cefixime and azithromycin. Compared with men who have sex with men (MSM), men who have sex with women only had higher odds of cefixime resistance (aOR 2.34, 95% CI 1.57–3.51; *p* < 0.001) and azithromycin resistance (aOR 2.21, 95% CI 1.18–4.17; *p* = 0.011), independent of travel history, antibiotic use, and STI co-infection (Appendix p8: Table S7).

### Gonorrhoea treatment

Among WHO EGASP cases in 2023 with positive gonococcal culture (n = 2502), the most frequent treatment was ceftriaxone (250–1000 mg) plus azithromycin (1–2 g) dual therapy (50%, 1250/2502). However, this treatment was only used in five of the countries: Cambodia 83% (239/274), the Philippines 51% (358/700), South Africa 73% (249/341), Thailand 42% (158/374), and Viet Nam 93% (246/260). In Thailand, ceftriaxone 500 mg and doxycycline dual therapy was the most common treatment (50%, 186/374) and in Zimbabwe all cases were treated with ceftriaxone 250 mg plus doxycycline. In Uganda, mainly cefixime 400 mg plus doxycycline was used (97%, 291/298), and Indonesia used cefixime 400 mg plus azithromycin 1 g for all cases. In Malawi, 99% (121/122) of cases were treated with gentamicin 240 mg plus doxycycline dual therapy.

## Discussion

Gonorrhoea remains a major public health concern globally, and antimicrobial-resistant *N. gonorrhoeae*, compromising gonorrhoea treatment, is becoming more prevalent internationally. In the present study, AMR in *N. gonorrhoeae* in 2023 within nine representative WHO EGASP sentinel countries in the WHO Western Pacific Region (Cambodia, the Philippines, Viet Nam), Southeast Asian Region (Indonesia, Thailand), and African Region (Malawi, South Africa, Uganda, Zimbabwe) is reported. Furthermore, demographic, behavioural, and clinical data of the gonorrhoea patients are described and associated with the gonococcal AMR. Briefly, the results elucidate high resistance levels to recommended gonorrhoea therapies, especially in the WHO Western Pacific Region. The high resistance rates for ceftriaxone, cefixime, and azithromycin were alarming in Cambodia and Viet Nam, and 9% (26/274) and 2% (4/260) of isolates in Cambodia and Viet Nam, respectively, was resistant to all three of these antimicrobials. In 2022, only four countries participated in the WHO EGASP, i.e., Cambodia, The Philippines, Thailand, and Uganda. Also in 2022, no resistance to ceftriaxone, cefixime or azithromycin was identified in The Philippines or Uganda. However, Thailand reported 0.4% (1/243 isolates) and 1.2% (3/243) resistance to ceftriaxone and cefixime, respectively. Furthermore, Cambodia reported 17.0% (32/188 isolates), 31.4% (59/188), and 6.9% (13/188) resistance to ceftriaxone, cefixime, and azithromycin, respectively.^27^ This may indicate that the resistance to ceftriaxone in Cambodia is relatively stable, but the resistance to cefixime and azithromycin continues to increase. The gonococcal AMR situation in Cambodia is imperative to continuously monitor in detail over longer time periods. Notably, also in another WHO Western Pacific Region country, i.e., China, the level of ceftriaxone resistance in *N. gonorrhoeae* is high and the resistance increased approximately three-fold from 2017 to 2022.^12^ Accordingly, in 2022 the China Gonococcal Resistance Surveillance Program (China-GRSP) identified a ceftriaxone resistance level of 8% (222/2804) in 13 provinces. The level of ceftriaxone resistance in the 13 provinces ranged from 0% (0/111) to 26% (14/53).^12^ Notably, treatment of sporadic ceftriaxone-resistant gonorrhoea cases^28^ and pharmacodynamic evaluations using a hollow fibre infection model for gonorrhoea^29^ have shown that ceftriaxone 1 g can cure most urogenital infections caused by ceftriaxone-resistant *N. gonorrhoeae* strains with ceftriaxone MICs of 0.25–1 mg/L, i.e., as all the 15% (42/274) and 20% (53/262) of ceftriaxone-resistant isolates cultured in Cambodia and Viet Nam, respectively, in 2023. All isolates in Indonesia, Malawi, the Philippines, Uganda, and Zimbabwe were in 2023 susceptible to ceftriaxone, cefixime, and azithromycin, and isolates in South Africa and Thailand only showed low prevalence of azithromycin resistance. Ciprofloxacin resistance was very high in all EGASP countries in which ciprofloxacin susceptibility was examined (n = 7). In univariable analysis, travelling within the country during the recent 30 days was associated with resistance to ceftriaxone, cefixime and azithromycin. Additionally, men who had sex with women only was associated with resistance to cefixime and azithromycin. All these associations remained in the multivariable analysis. Nevertheless, these associations were mainly based on the patients with gonorrhoea in Cambodia and Viet Nam, where all resistance to ceftriaxone and cefixime, and most azithromycin resistance, was detected. Accordingly, in Cambodia and Viet Nam internal mobility may facilitate dissemination of AMR gonococcal strains, but the associations can also reflect access to care and treatment-seeking behaviour patterns not captured in the epidemiological variables of WHO EGASP. The increased resistance to cefixime and azithromycin observed among men who had sex with women only compared with MSM may reflect differential healthcare access, testing frequency, or under-surveilled transmission within heterosexual sexual networks. These observations underscore the importance of including also heterosexual men in targeted surveillance and AMR prevention efforts, which have historically focused on MSM due to higher testing rates and STI burden in that population. Our results also underscore the need for genomic and network-based approaches to better resolve the underlying gonococcal transmission dynamics associated with observed phenotypic AMR patterns.

*N. gonorrhoeae* is one of the WHO priority AMR pathogens categorised for urgency that pose high threat to human public health, therefore global, regional, and national actions are necessary.^22^^,^^23^^,^^30^ The vast majority of ceftriaxone-resistant cases has currently been identified in or linked to Asia, but travellers facilitate the national and international spread of ceftriaxone-resistant *N. gonorrhoeae* strains and ultimately these strains will initiate endemic spread in other regions worldwide.^2^^,^^5^, ^6^, ^7^^,^^9^, ^10^, ^11^, ^12^, ^13^, ^14^, ^15^, ^16^, ^17^, ^18^, ^19^ Accordingly, global surveillance of gonococcal AMR is imperative.^2^^,^^5^, ^6^, ^7^, ^8^, ^9^^,^^14^, ^15^, ^16^, ^17^, ^18^, ^19^^,^^31^^,^^32^ The WHO EGASP surveillance involves national and international multidisciplinary experts, and the results indicate that national trend estimations and comparisons within and across regions are feasible, despite the limited data from only sentinel countries. The standardised and quality-assured WHO EGASP data have been effectively used in several countries; to identify high-risk groups, trends in gonococcal AMR and associations with gonorrhoea patients, and, through the participating Ministries of Health, to refine national management guidelines and public health policies and strategies.

The limitations of the WHO EGASP include the limited number of participating sentinel countries and gonococcal isolates examined. The low level of reporting of some critical epidemiological variables, such as sexual orientation, sexual practices and self-reporting of HIV status and STI co-infections (which are most frequently not tested for aetiological diagnosis), is also a limitation. The WHO EGASP is also confined to males and was in 2023 not including extragenital samples. Finally, no data on ethnicity is collected in the WHO EGASP.

Nevertheless, the WHO EGASP is continuously working on addressing these limitations and the programme has been substantially strengthened since 2015. Firstly, the standardised and quality-assured EGASP surveillance has been expanded from one country (Thailand) to nine countries representing three WHO regions. The number of intra-country sentinel clinical sites have also expanded substantially. Furthermore, Argentina, Brazil, Cote D'Ivoire, India, and Qatar are currently in different stages of EGASP implementation. Secondly, the WHO has published updated global treatment recommendations for STIs, including recommending ceftriaxone 1 g monotherapy for treatment of gonorrhoea,^33^ and revisions of the national STI management guidelines in several WHO EGASP countries (Cambodia, Indonesia, South Africa, Viet Nam, and the Philippines) have been finalised or initiated. Thirdly, in 2024, a global external quality assessment (EQA) scheme was launched for WHO EGASP and the 2024 WHO *N. gonorrhoeae* reference strain panel was published to improve internal quality control and EQA.^26^ Fourthly, WHO EGASP supplementary protocols for whole-genome sequencing (WGS), extra-genital (anorectum and oropharynx) sampling, and test-of-cure^21^ have been published and implemented, where feasible. Fifthly, WGS training has been provided at the WHO Collaborating Centre for Gonorrhoea and Other STIs in Sweden for representatives from Malawi, the Philippines, South Africa, Thailand, Uganda, and Viet Nam. Lastly, WHO EGASP studies have been performed to examine the susceptibility to the promising novel antimicrobials zoliflodacin (https://gardp.org/positive-results-announced-in-largest-pivotal-phase-3-trial-of-a-first-in-class-oral-antibiotic-to-treat-uncomplicated-gonorrhoea/)^34^ and gepotidacin,^35^ as well as tetracycline, i.e., to estimate potential impact of doxycycline post-exposure prophylaxis on gonorrhoea.^36^

In conclusions, WHO EGASP is a critical international programme that reports gonococcal AMR and important demographic, behavioural and clinical data of gonorrhoea patients; and supports the strengthening of gonorrhoea and AMR surveillance for *N. gonorrhoeae* across WHO regions. In 2023, we report most alarming levels of resistance to ceftriaxone, cefixime and azithromycin in the WHO Western Pacific Region countries Cambodia and Viet Nam. Generated WHO EGASP data contribute to the refinements of international and national STI treatment guidelines, laboratory diagnostic standards, and review of health workers' skills at the national level. WHO EGASP is continuously expanding by including additional countries, across WHO regions, and sentinel clinics, within countries, as well as improving the programme. For example, recently a global EGASP EQA, harmonised AMR breakpoints, extragenital sampling, WGS and test-of-cure have been implemented, where feasible. Ultimately, novel antimicrobials, such as zoliflodacin^34^ and gepotidacin,^35^ and/or a gonococcal vaccine will be required for effective global management and control of gonorrhoea.

## Contributors

IM, MU, and TW designed, initiated, and coordinated the study. The WHO EGASP country representatives coordinated patient recruitment, data collection, culture of clinical samples, and antimicrobial susceptibility testing in their countries. IM, PV, and MU analysed and interpreted all the results and wrote a first draft of the paper. All coauthors and study group members read, commented on, and approved the final manuscript and submission of the manuscript. The first author (IM), second author (PV) and both corresponding authors (MU and TW) had full access to and verified all the data in the study and had final responsibility for the decision to submit for publication.

## Data sharing statement

Most data are presented in the present paper. Additional data may be made available from the corresponding author upon reasonable request.

## Declaration of interests

All authors report no conflicts of interest.

## Appendix A Supplementary data

Appendix

EGASP Study Group
