# Supplementary material for: Antimicrobial resistance in Neisseria gonorrhoeae in nine sentinel countries within the World Health Organization Enhanced Gonococcal Antimicrobial Surveillance Programme (EGASP), 2023: a retrospective observational study
Source: Lancet Reg Health West Pac. 2025 Aug 21;61:101663. doi: 10.1016/j.lanwpc.2025.101663 (PMC12414356; doi:10.1016/j.lanwpc.2025.101663)
Supplement: Appendix [file mmc1.docx]

***Table S1***: The 38 clinical sentinel sites and 17 laboratories participating in the World Health Organization (WHO) Enhanced Gonococcal Antimicrobial Surveillance Programme (EGASP) in 2023

| WHO Region | Country | City | Sentinel site | Collection start date | Collection end date | Reference laboratory |
| --- | --- | --- | --- | --- | --- | --- |
| Africa | **Malawi** | Lilongwe | Bwaila clinic | 10/9/2023 | 12/19/2023 | Tidziwe Clinic (UNC Project) |
|  | **South Africa** | Johannesburg | Alexandra Health Centre | 2/21/2023 | 11/27/2023 | National Institute for Communicable Diseases |
|  |  | Durban | Prince Cyril Zulu Communicable Diseases Centre | 2/7/2023 | 11/2/2023 |  |
|  |  | Cape Town | Spencer Road Clinic | 1/25/2023 | 12/7/2023 |  |
|  | **Uganda** | Kampala | Naguru Teenage Health and Information Center (key teenage-focused health-care facility) | 1/11/2023 | 10/26/2023 | Translational Laboratory Infectious Diseases Institute – Makerere University |
|  |  |  | Kisenyi Health Centre IV (densely populated facility) | 1/3/2023 | 6/14/2023 | National Microbiology Reference Laboratory, Central Public Health Laboratories, Ministry of Health |
|  |  |  | Most at Risk Populations Initiative Clinic (focused on key populations) | 1/16/2023 | 12/13/2023 |  |
|  | **Zimbabwe** | Bulawayo | Khami Road Clinic | 10/25/2023 | 12/20/2023 | National Microbiology Reference Laboratory (NMRL) |
|  |  |  | Cowdry Park Clinic | 10/24/2023 | 12/12/2023 |  |
|  |  | Beitbridge | Dulibadzimu Clinic | 10/31/2023 | 12/14/2023 |  |
| Western Pacific | **Cambodia** | Phnom Penh | Family Health Clinic (FHC) | 1/4/2023 | 9/16/2023 | National Institute of Public Health |
|  |  |  | FHC Samdach Ov | 1/4/2023 | 9/16/2023 |  |
|  |  |  | FHC Chactomok | 5/9/2023 | 9/20/2023 |  |
|  |  |  | FHC Pochentong | 3/13/2023 | 9/20/2023 |  |
|  |  |  | FHC Turl Kok | 1/14/2023 | 9/22/2023 |  |
|  |  |  | National Clinic for Dermatology and STDs | 1/30/2023 | 6/12/2023 |  |
|  |  |  | Chhouk Sar Clinic | 1/10/2023 | 9/22/2023 |  |
|  |  | Kampong Speu | FHC in Kampong Speu Regional Hospital | 5/11/2023 | 8/11/2023 |  |
|  |  | Prey Veng | FHC in Prey Veng Regional Hospital | 2/27/2023 | 9/5/2023 |  |
|  |  |  | FHC Neak Leong | 1/27/2023 | 9/11/2023 |  |
|  |  | Kampong Cham | FHC in Prey Veng Regional Hospital | 1/4/2023 | 9/13/2023 |  |
|  | **The Philippines** | Metro Manila | SACCL-SLH Out-Patient Department Clinic | 1/3/2023 | 3/28/2023 | SACCL-SLH Out-Patient Department Clinic |
|  |  |  | Manila Social Hygiene Clinic | 1/11/2023 | 3/27/2023 |  |
|  |  |  | Taguig Social Hygiene Clinic & Drop-In Center | 1/4/2023 | 11/29/2023 |  |
|  |  |  | Paranaque Social Hygiene Clinic & Wellness Center | 1/3/2023 | 11/29/2023 |  |
|  |  |  | LoveYourself Community Center – Victoria | 1/3/2023 | 11/24/2023 | Research Institute of Tropical Medicine |
|  |  | Davao | Davao Reproductive Health and Wellness Center | 1/3/2023 | 11/16/2023 | Southern Philippines |
|  |  | Cebu | Cebu Social Hygiene Clinic | 1/9/2023 | 6/5/2023 | Vicente Sotto Memorial Medical Center |
|  | **Viet Nam** | Hanoi | National Hospital of Dermatology and Venereology | 6/3/2023 | 9/21/2023 | National Hospital of Dermatology and Venereology |
|  |  | Ho Chi Minh City | HCMC Dermatology and Venereology Hospital | 8/7/2023 | 9/21/2023 | HCMC Dermatology and Venereology Hospital |
|  |  | Quy Hoa | Quy Hoa Central Hospital for Leprosy and Dermato-Venereology | Collection started in 2024 |  | Quy Hoa Central Hospital for Leprosy and Dermato-Venereology |
| South-East Asia | **Indonesia** | Jakarta | Klinik Globalindo | 10/3/2023 | 12/6/2023 | Sulianti Saroso Hospital |
|  |  |  | Puskesmas Koja | 10/11/2023 | 11/29/2023 |  |
|  |  |  | Puskesmas Kebon Jeruk | 10/9/2023 | 12/7/2023 |  |
|  |  |  | Puskesmas Kebayoran Baru | 10/10/2023 | 12/8/2023 |  |
|  | **Thailand** | Bangkok | Bangrak Hospital | 3/1/2023 | 12/28/2023 | Bangrak Hospital |
|  |  |  | Silom Community Clinic | 3/1/2023 | 12/4/2023 | Clinic Faculty of Tropical Medicine |
|  |  | Chiang Mai | ODPC 1st | 4/1/2023 | 12/25/2023 | ODPC 1^st^ |

***Table S2:* Patient characteristics and exposure factors, WHO EGASP, 2023**

|  | **Cambodia** | **Indonesia** | **Malawi** | **Philippines** | **South Africa** | **Thailand** | **Uganda** | **Viet Nam** | **Zimbabwe*** | **Overall** |
| --- | --- | --- | --- | --- | --- | --- | --- | --- | --- | --- |
| **Urethral discharge episodes** | **380** | **126** | **126** | **786** | **528** | **762** | **423** | **260** | **107** | **3498** |
| **Age** (years) | **N (%)** | **N (%)** | **N (%)** | **N (%)** | **N (%)** | **N (%)** | **N (%)** | **N (%)** | **N (%)** | **N (%)** |
| <18 | 0 (0) | 0 (0) | 0 (0) | 44 (6) | 0 (0) | 25 (3) | 12 (3) | 3 (1) | 0 (0) | 84 (2) |
| 18-24 | 171 (45) | 51 (40) | 27 (21) | 419 (53) | 79 (15) | 225 (30) | 142 (34) | 84 (32) | 27 (25) | 1225 (35) |
| 25 to 34 | 141 (37) | 61 (48) | 67 (53) | 266 (34) | 263 (50) | 229 (30) | 136 (32) | 124 (48) | 42 (39) | 1327 (38) |
| 35 to 44 | 58 (15) | 12 (1) | 23 (18) | 44 (6) | 140 (27) | 118 (15) | 86 (20) | 38 (15) | 27 (25) | 549 (16) |
| ≥45 | 10 (3) | 2 (2) | 9 (7) | 13 (2) | 46 (9) | 165 (22) | 47 (11) | 11 (4) | 9 (8) | 312 (9) |
| Median [IQR] | 25 [21, 32] | 26 [22, 29] | 29 [25, 35] | 23 [21, 28] | 32 [27, 37] | 29 [23, 42] | 28 [23, 37] | 28 [23, 33] | 29 [24, 38] | 27 (22, 34) |
| Range | 15-57 | 18-54 | 18-63 | 12-62 | 18-61 | 13-93 | 16-65 | 14-64 | 18-59 | 12-93 |
| **Antibiotic use** *(recent two weeks)* |  |  |  |  |  |  |  |  |  |  |
| Yes | 5 (1) | 24 (19) | 2 (2) | 91 (12) | 7 (1) | 168 (22) | 77 (18) | 7 (3) | 4 (4) | 385 (11) |
| No | 326 (86) | 99 (79) | 124 (98) | 695 (88) | 484 (92) | 574 (75) | 335 (79) | 217 (83) | 102 (95) | 2956 (84) |
| Unknown | 49 (13) | 3 (2) | 0 (0) | 0 (0) | 37 (7) | 20 (3) | 11 (3) | 36 (14) | 1 (1) | 157 (4) |
| **History of travel** *(recent 30 days)* |  |  |  |  |  |  |  |  |  |  |
| Yes | 163 (43) | 60 (48) | 20 (16) | 63 (8) | - | - | 6 (1) | 184 (71) | 37 (35) | 533 (15) |
| *Within country Only* | 162 (42) | 55 (44) | 17 (13) | 51 (6) | - | - | 0 (0) | 178 (68) | 27 (25) | 490 (14) |
| *Internationally* | 0 (0) | 3 (2) | 3 (2) | 12 (2) | - | - | 6 (1) | 3 (1) | 8 (7) | 35 (1) |
| *Both* | 1 (0.3) | 2 (2) | 0 (0) | 0 (0) | - | - | 0 (0) | 3 (1) | 2 (2) | 8 (0.2) |
| No | 217 (57) | 66 (52) | 106 (84) | 723 (92) | - | - | 417 (99) | 74 (28) | 69 (64) | 1672 (48) |
| Unknown | 0 (0) | 0 (0) | 0 (0) | 0 (0) | 528 (100) | 762 (100) | 0 (0) | 2 (1) | 1 (1) | 1290 (37) |
| **Sexual partners** *(recent 30 days)* |  |  |  |  |  |  |  |  |  |  |
| 1 | 169 (44) | 87 (69) | 67 (53) | 437 (56) | 252 (48) | - | 218 (52) | 148 (57) | 42 (39) | 1420 (41) |
| 2-5 | 206 (54) | 36 (29) | 58 (46) | 296 (38) | 241 (46) | - | 188 (44) | 71 (27) | 48 (45) | 1144 (33) |
| ≥6 | 5 (1) | 2 (2) | 1 (1) | 52 (7) | 3 (1) | - | 15 (4) | 1 (0.4) | 2 (2) | 81 (2) |
| Unknown | 0 (0) | 1 (1) | 0 (0) | 1 (0.1) | 32 (6) | 762 (100) | 2 (0.5) | 40 (15) | 15 (14) | 853 (24) |

WHO=World Health Organization; EGASP=Enhanced Gonococcal Antimicrobial Susceptibility Programme; N=Number; IQR=Inter-quartile range

*Two participants from Zimbabwe did not report age.

***Table S3:* Patient sexual history, practices and co-infections, WHO EGASP, 2023**

|  | **Cambodia** | **Indonesia** | **Malawi** | **Philippines** | **South Africa** | **Thailand** | **Uganda** | | **Viet Nam** | | **Zimbabwe** | **Overall** |
| --- | --- | --- | --- | --- | --- | --- | --- | --- | --- | --- | --- | --- |
|  | **N (%)** | **N (%)** | **N (%)** | **N (%)** | **N (%)** | **N (%)** | **N (%)** | | **N (%)** | | **N (%)** | **N (%)** |
| **Urethral discharge episodes** | **380** | **126** | **126** | **786** | **528** | **762** | **423** | | **260** | | **107** | **3498** |
| **Sexual history** (previous 30 days) | | | | | | | | | | | | |
| Sex with women only | 244 (64) | 84 (67) | – | 356 (45) | 526 (99) | 538 (71) | – | | 230 (88) | | 106 (99) | 2084 (60) |
| Sex with men only | 97 (26) | 37 (29) | – | 314 (40) | 2 (0.4) | 201 (26) | – | | 25 (9) | | 0 (0) | 676 (19) |
| Sex with men and women | 39 (10) | 5 (4) | – | 116 (15) | 0 (0) | 12 (2) | – | | 4 (2) | | 0 (0) | 176 (5) |
| Unknown | – | – | 126 (100) | - | - | 11 (1) | 423 (100) | | 1 (0.4) | | 1 (1) | 562 (16) |
| **Sexual practices** (previous 30 days; multiple responses possible) | | | | | | | | | | | | |
| Vaginal sex | 286 (75) | 88 (70) | – | 364 (46) | – | – | – | | 222 (86) | | 104 (97) | 1064 (64) |
| Anal sex | 139 (36) | 40 (32) | – | 522 (66) | – | – | – | | 36 (14) | | – | 737 (44) |
| Anal receptive | 134 (35) | 18 (14) | – | 362 (46) | – | – | – | | 15 (6) | | – | 529 (32) |
| Anal insertive | – | 32 (25) | – | 160 (20) | – | – | – | | 31 (12) | | – | 223 (13) |
| Oral sex | 6 (2) | 31 (24) | – | 537 (68) | – | – | – | | 161 (62) | | 2 (2) | 731 (44) |
| Oral receptive | – | 16 (13) | – | 345 (44) | – | – | – | | 34 (13) | | 1 (1) | 396 (24) |
| Oral insertive | 6 (2) | 15 (12) | – | 192 (24) | – | – | – | | 128 (49) | | 1 (1) | 342 (21) |
| **Positive HIV status** (self-report) | |  |  |  |  |  | |  | |  | |  |
| Yes | – | 15 (12) | 5 (4) | 36 (5) | – | – | 24 (6) | | 5 (2) | | 14 (13) | 99 (3) |
| No | 0 (0) | 5 (4) | 69 (55) | 5 (1) | – | – | 0 (0) | | 176 (68) | | 78 (73) | 333 (10) |
| Unknown | 380 (89) | 106 (84) | 52 (41) | 745 (95) | 528 (100) | 762 (100) | 399 (94) | | 79 (30) | | 15 (14) | 3066 (88) |
| **Concurrent STI** (other than HIV, self-report) | | | | | |  | | | | | | |
| Yes | 50 (13) | 22 (17) | 59 (47) | 62 (8) | – | – | 24 (6) | | 24 (9) | | 17 (16) | 254 (7) |
| No | 330 (87) | 104 (83) | 67 (53) | 724 (92) | – | – | 327 (77) | | 212 (82) | | 90 (84) | 1184 (53) |
| Unknown | – | – | – | – | 528 (100) | 762 (100) | 72 (17) | | 24 (8) | | – | 1386 (40) |

***Table S4:* Univariable association of ceftriaxone resistance (MIC>0.125 mg/L) and patient characteristics, WHO EGASP, 2023**

|  | **Ceftriaxone resistance**  **% (n/N)** | **Odds ratio** | **95% CI** | ***p*-value** |
| --- | --- | --- | --- | --- |
|  |  |  |  |  |
| **Age** (years, n = 2491) |  |  |  |  |
| <25 | 3.8 (39/1028) | 1 |  |  |
| >25 | 3.8 (56/1463) | 1.04 | 0.69-1.59 | 0.85 |
| **Antibiotic use** (previous two weeks, n = 2381) | | | | |
| No | 3.3 (72/2155) |  |  |  |
| Yes | 2.2 (5/226) |  |  |  |
| **History of internal travel** (previous 30 days, n = 1777) | | | | |
| No | 3.0 (40/1345) | 1 |  |  |
| Yes | 12.5 (54/432) | 4.66 | 3.06-7.16 | <0.001^b^ |
| **Sexual history** (previous 30 days, n = 2065) | | | | |
| Men who have sex with men^a^ | 1.0 (7/672) |  |  |  |
| Men who had sex with women only | 6.3 (88/1393) |  |  |  |
| **Number of sexual partners** (previous 30 days, n = 2044) | | | | |
| Single | 4.7 (50/1063) | 1 |  |  |
| Multiple | 3.7 (36/981) | 0.77 | 0.50-1.19 | 0.25 |
| **HIV status** (self-report, n = 412) | | | | |
| HIV positive | 0 (0/103) |  |  |  |
| HIV negative | 12.3 (38/309) |  |  |  |
| **Concurrent STI infection** (self-report/syndromic diagnosis, n= 1710) | | | | |
| Yes | 2.6 (4/153) |  |  |  |
| No | 5.5 (85/1557) |  |  |  |
| n=number of patients providing a specific answer; N=number of patients reporting on the variable  ^a^ The variable “men who have sex with men” includes those participants who reported having sex with men only and those who mentioned that they had sex with both men and women.  ^b^ *p<*0.05 represents statistical significance. | | | | |

***Table S5:* Univariable association of cefixime resistance (MIC>0.125 mg/L) and patient characteristics, WHO EGASP, 2023**

|  | **Cefixime resistance** | **Odds ratio** | **95% CI** | ***p*-value** |
| --- | --- | --- | --- | --- |
|  | **% (n/N)** |  |  |  |
| **Age** (years, n = 2332) |  |  |  |  |
| <25 | 9.8 (94/958) | 1 |  |  |
| >25 | 9.3 (128/1374) | 0.94 | 0.71-1.25 | 0.74 |
| **Antibiotic use** (previous two weeks, n = 2222) | | | | |
| Yes | 3.1 (6/196) |  |  |  |
| No | 8.7 (177/2026) |  |  |  |
| **History of internal travel** (previous 30 days, n = 1774) | | | | |
| No | 8.0 (108/1346) | 1 |  |  |
| Yes | 26.4 (113/428) | 4.11 | 3.07-5.50 | <0.001^b^ |
| **Sexual history** (previous 30 days, n = 2059) | | | | |
| Men who have sex with men^a^ | 5.4 (36/672) | 1 |  |  |
| Men who had sex with women only | 14.2 (197/1387) | 2.92 | 2.05-4.29 | <0.001^b^ |
| **Number of sexual partners** (previous 30 days, n = 2041) | | | | |
| Single | 9.1 (97/1061) | 1 |  |  |
| Multiple | 11.3 (111/980) | 1.27 | 0.95-1.69 | 0.10 |
| **HIV status** (self-report, n = 412) | | | | |
| HIV positive | 1.0 (1/103) |  |  |  |
| HIV negative | 17.5 (54/309) |  |  |  |
| **Concurrent STI infection** (self-report, n= 1707) | | | | |
| Yes | 4.6 (7/153) |  |  |  |
| No | 13.3 (206/1554) |  |  |  |
| n=number of patients providing a specific answer; N=number of patients reporting on the variable  ^a^ The variable “men who have sex with men” includes those participants who reported having sex with men only and those who mentioned that they had sex with both men and women.  ^b^ *p<*0.05 represents statistical significance. | | | | |

***Table S6:* Univariable association of azithromycin resistance (MIC>1.0 mg/L) and patient characteristics, WHO EGASP, 2023**

|  | **Azithromycin resistance** | **Odds** **ratio** | **95% CI** | ***p*-value** |
| --- | --- | --- | --- | --- |
|  | **% (n/N)** |  |  |  |
| **Age** (years, n = 2491) |  |  |  |  |
| <25 | 3.5 (36/1033) | 1 |  |  |
| >25 | 3.6 (53/1458) | 1.05 | 0.68-1.60 | 0.60 |
| **Antibiotic use** (past two weeks, n = 2380) | | | | |
| No | 3.4 (74/2154) |  |  |  |
| Yes | 1.3 (3/226) |  |  |  |
| **History of internal travel** (past 30 days, n = 1777) | | | | |
| No | 3.5 (47/1345) | 1 |  |  |
| Yes | 6.7 (29/432) | 1.99 | 1.22-3.18 | <0.001^b^ |
| **Sexual history** (past 30 days, n = 2064) | | | | |
| Men who have sex with men^a^ | 3.1 (21/672) | 1 |  |  |
| Men who had sex with women only | 4.9 (68/1392) | 2.57 | 1.39-5.20 | <0.001^b^ |
| **Number of sexual partners** (past 30 days, n = 2043) | | | | |
| Single | 4.2 (45/1062) | 1 |  |  |
| Multiple | 4.1 (40/981) | 0.96 | 0.62-1.48 | 0.86 |
| **HIV status** (self-report, n = 412) | | | | |
| HIV positive | 1.0 (1/103) |  |  |  |
| HIV negative | 4.5 (14/309) |  |  |  |
| **Concurrent STI infection** (self-report, n= 1710) | | | | |
| Yes | 1.3 (2/153) |  |  |  |
| No | 4.7 (73/1557) |  |  |  |
| n=number of patients providing a specific answer; N=number of patients reporting on the variable  ^a^ The variable “men who have sex with men” includes those participants who reported having sex with men only and those who mentioned that they had sex with both men and women.  ^b^ *p<*0.05 represents statistical significance. | | | | |

***Table S7:* Univariable (crude OR) and multivariable (adjusted OR) logistic regression of patient characteristics associated with resistance to ceftriaxone (MIC>0.125 mg/L), cefixime (MIC>0.125 mg/L), or azithromycin (MIC>1.0 mg/L)**

| **Exposure**  **Variable** | **Antimicrobial** | **Crude OR (95% CI)** | ***p*-value^b^** | **Adjusted OR (95% CI)** | ***p*-value^b^** |
| --- | --- | --- | --- | --- | --- |
| **History of internal travel** | Ceftriaxone | 4.66 (3.06-7.16) | <0.001 | 4.12 (2.65-6.65) | <0.001 |
|  | Cefixime | 4.11 (3.07-5.50) | <0.001 | 3.75 (2.62-5.37) | <0.001 |
|  | Azithromycin | 1.99 (1.22-3.18) | <0.001 | 1.73 (1.03-2.91) | 0.043 |
| **Sexual history^a^** | Cefixime | 2.92 (2.05-4.29) | <0.001 | 2.34 (1.57-3.51) | <0.001 |
|  | Azithromycin | 2.57 (1.39-5.20) | <0.001 | 2.21 (1.18-4.17) | 0.011 |

OR=odds ratio; CI=confidence interval; NA=not applicable

^a^ Men who have sex with women only versus men who have sex with men.

^b^ *p<*0.05 represents statistical significance.

^†^**WHO EGASP study group**

Cambodia: Lon Say Heng, Vichea Ouk, Mot Virak, Phal Kun Mom, Serongkea Deng; Indonesia: Vivi Setiawaty, Endang Lukitosari, Nurhalina Afriana, Verawati Sulaiman, Teguh S. Hartono, Maria Laurensia, Ni Luh Putu Pitawati; Malawi: Mitch Matoga, Irving Hoffman, Robert Krysiak, Jane Chen, Naomi Bonongwe, Claightone Chirombo, Edward Jere, James Kapha; The Philippines: Sonia B. Sia, Manuel C Jr. Jamoralin, Marietta Lagrada, June Gayeta, Jaywardeen Abad, Noel Palaypayon, Diana Lim, Iftizar N. Haron, Joseph Carlo Sangco, Felyrose Fuertes, August Cesar Abrajano, Ruby Rusia-Uy, Christine Ivy Paula S. Agtuca, Ma. Theresa A. Fedoc-Minguito, Louwela A. Jerusalem; South Africa: Venessa Maseko, Etienne Müller, Lindy Gumede, Portia Baloyi; Thailand: Rossaphorn Kittiyaowamarn, Natnaree Girdthep, Porntip Paopang, Pongsathorn Sangprasert, Thitima Cherdtrakulkiat, Jaray Tongtoyai; Uganda: Francis Kakooza, Peter Kyambadde, Emmanuel Mande, Martha Nakasi; Vietnam: **Le Huu Doanh,** Pham Thi Lan, Pham Quynh Hoa, Pham Dieu Hoa, Thuy Thi Phan Nguyen, Hao Trong Nguyen, Nhi Thi Uyen Pham, Phuong Thi Thanh Nguyen; University of Washington: Francis Slaughter; WHO Vietnam: Nguyen Thi Thuy Van; Zimbabwe: Anna Machiha, Owen Mugurungi, Agnes Juru, Tatenda Ngorima, Lucia Sisya, Kudzai Takarinda, Andrew Tarupiwa; WHO Zimbabwe: Muchaneta Mugabe, Mkhokheli Ngwenya, Precious Paidamoyo Andifasi; WHO CC-Australia: Monica Lahra, Sebastian van Hal; WHO CC-Sweden: Magnus Unemo, Daniel Golparian, Susanne Jacobsson, Daniel Schröder; WHO HQ: Teodora Wi, Ismael Maatouk, Phiona Vumbugwa
