# Supplementary material for: Antimicrobial resistance in Neisseria gonorrhoeae in nine sentinel countries within the World Health Organization Enhanced Gonococcal Antimicrobial Surveillance Programme (EGASP), 2023: a retrospective observational study
Source: Lancet Reg Health West Pac. 2025 Aug 21;61:101663. doi: 10.1016/j.lanwpc.2025.101663 (PMC12414356; doi:10.1016/j.lanwpc.2025.101663)
Supplement: EGASP Study Group [file mmc2.docx]

| **First names** | **Surname** |
| --- | --- |
| Lon Say | Heng |
| Vichea | Ouk |
| Mot | Virak |
| Phal Kun | Mom |
| Serongkea | Deng |
| Vivi | Setiawaty |
| Endang | Lukitosari |
| Nurhalina | Afriana |
| Verawati | Sulaiman |
| Teguh S. | Hartono |
| Maria | Laurensia |
| Ni Luh Putu | Pitawati |
| Mitch | Matoga |
| Irving | Hoffman |
| Robert | Krysiak |
| Jane | Chen |
| Naomi | Bonongwe |
| Claightone | Chirombo |
| Edward | Jere |
| James | Kapha |
| Sonia B. | Sia |
| Manuel C Jr. | Jamoralin |
| Marietta | Lagrada |
| June | Gayeta |
| Jaywardeen | Abad |
| Noel | Palaypayon |
| Diana | Lim |
| Iftizar N. | Haron |
| Joseph Carlo | Sangco |
| Felyrose | Fuertes |
| August Cesar | Abrajano |
| Ruby | Rusia-Uy |
| Christine Ivy Paula S. | Agtuca |
| Ma. Theresa A. | Fedoc-Minguito |
| Louwela A. | Jerusalem |
| Venessa | Maseko |
| Etienne | Müller |
| Lindy | Gumede |
| Portia | Baloyi |
| Rossaphorn | Kittiyaowamarn |
| Natnaree | Girdthep |
| Porntip | Paopang |
| Pongsathorn | Sangprasert |
| Thitima | Cherdtrakulkiat |
| Jaray | Tongtoyai |
| Francis | Kakooza |
| Peter | Kyambadde |
| Emmanuel | Mande |
| Martha | Nakasi |
| **Le Huu** | **Doanh** |
| Pham Thi | Lan |
| Pham Quynh | Hoa |
| Pham Dieu | Hoa |
| Thuy Thi Phan | Nguyen |
| Hao Trong | Nguyen |
| Nhi Thi Uyen | Pham |
| Phuong Thi Thanh | Nguyen |
| Nguyen Thi Thuy | Van |
| Francis | Slaughter |
| Anna | Machiha |
| Owen | Mugurungi |
| Agnes | Juru |
| Tatenda | Ngorima |
| Lucia | Sisya |
| Kudzai | Takarinda |
| Andrew | Tarupiwa |
| Muchaneta | Mugabe |
| Mkhokheli | Ngwenya |
| Precious Paidamoyo | Andifasi |
| Monica | Lahra |
| Sebastian | van Hal |
| Magnus | Unemo |
| Daniel | Golparian |
| Susanne | Jacobsson |
| Daniel | Schröder |
| Teodora | Wi |
| Ismael | Maatouk |
| Phiona | Vumbugwa |
